# How are European birth-cohort studies engaging and consulting with young cohort members?

**DOI:** 10.1186/1471-2288-13-56

**Published:** 2013-04-11

**Authors:** Patricia J Lucas, Debra Allnock, Tricia Jessiman

**Affiliations:** 1 School for Policy Studies, University of Bristol, 8 Priory Rd, Bristol BS8 1TZ, UK

## Abstract

**Background:**

Birth cohort studies, where parents consent for their child to be enrolled in a longitudinal study prior to or soon after birth, are a powerful study design in epidemiology and developmental research. Participation often continues into adulthood. Where participants are enrolled as infants, provision should be made for consent, consultation and involvement in study design as they age. This study aims to audit and describe the extent and types of consultation and engagement currently used in birth cohorts in Europe.

**Methods:**

Seventy study groups (representing 84 cohorts) were contacted to ask about their practice in engaging and involving study members. Information was gathered from study websites and publications, 15 cohorts provided additional information via email and 17 cohorts were interviewed over the phone.

**Results:**

The cohorts identified confirm the growth of this study design, with more than half beginning since 1990, and 4 since 2011. Most studies maintain a website open to the general public, although many are written for the scientific community only. Five studies have web pages specifically for young cohort members and one study provides a dedicated page for fathers. Cohorts send newsletters, cards, and summaries of findings to participants to stay in touch. Six cohorts use Facebook for this purpose. Five cohorts provide feedback opportunities for participants after completing a round of data collection. We know of just 8 cohorts who have a mechanism for consulting with parents and 3 a mechanism for consulting with young people themselves, although these were ‘one off’ consultations for some groups. Barriers to further consultation with cohort members were: concerns about impact on quality of research, ethical constraints, resource limitations, lack of importance, and previous adverse experiences.

**Conclusions:**

Although the children in some of the cohorts are still young (born in the last 10 years) many are old enough to include some element of consultation. Barriers to greater participation identified here have been overcome in some cohorts and in other fields. Within the scope of their funding and resources, birth cohort studies should consider ways in which they could increase engagement, consultation, and co-production with research participants.

## Background

It is argued that greater involvement of the public in research leads to better research outcomes through improvements to data quality, data collection, and, in time, dissemination [1-6]. This may be particularly true for children and young people because of the likely greater difference between their experiences and those of adult researchers. Issues relevant to young people may be overlooked by adult researchers [7], and data collection tools which are disliked or poorly understood by participants are more likely to be returned incomplete or incorrect. As co-researchers, young people can elicit better data from other young people or facilitate better access to respondents [8]. In dissemination, young people may advise on the accessibility of publications to other young people and in public events can have a greater impact on audiences than adult researchers [8,9]. Considering ethical practice, researchers must consider when and how to consent children, and consultation with them will improve this process [10,11]. The small literature that documents changes resulting from greater involvement of the public and participants [2,12,13] suggests research improvements, although more evidence is needed.

As well as the potential benefits to research, greater involvement of children and young people has a rights-based dimension. The United Nations Convention on the Rights of the Child includes the right for children to express their views freely in all matters affecting them (Article 12) and for those views to be given due weight in accordance with the age and maturity of the child [14]. Taken further, children’s involvement might be seen as emancipatory; sharing power between the researched (who are a frequently powerless group) and the researcher [9]. There may also be opportunities for children and young people themselves to benefit from research participation; they may gain skills and knowledge through their involvement that will benefit them in later life.

Involving the public in research is also a priority for politicians and funding bodies in health [3,15]. Methods for involving adult research participants and service users are well developed in some fields [16-19]. Methods for involving children and families are less present. Although good practice exists including in child-led research [20-22], practice in this field is often restricted to service users and qualitative research. There is no intrinsic reason why quantitative research should not also engage in co-production.

Within longitudinal studies, such as birth cohorts, a relationship between researcher and researched must be sustained for many years. Retaining members in the study over the long term is crucial for the study to succeed, but attrition is often a problem [23]. For those convinced of the value of participation, this would seem like the natural home for building a more participatory relationship. Researchers benefit by increased engagement, completion, and (potentially) retention in the study. Study members benefit by having an opportunity to contribute to the research in which they are a part. Birth cohorts (where recruitment is instigated prior to or soon after birth and participants are followed for several years) are currently accorded a high priority in public health research [24]. This interest is evidenced by, for example, the major investment by the ESRC/MRC in the UK Birth Cohort Study and associated large scale facility project [25], and the US National Children’s Study [26]. As well as the necessity of a strong relationship with cohort members, these study types have another advantage for those who advocate participation in the potential created by ongoing data collection for research to respond to the changing world experienced by research participants. Important environmental contexts for health (e.g. trends in use of social media, multi-channel screen time, types and availability of illicit drugs) will likely be better understood in the first instance by those experiencing these changes than by those researching them.

While arguments for participant involvement are well rehearsed, the extent of participant involvement in research practice is often unknown. The study reported here aims to audit and describe the extent and types of consultation, engagement and participation currently used in one type of longitudinal study (birth cohorts) in Europe. We were particularly interested in methods to increase engagement of young cohort members, including but not restricted to opportunities to contribute to research planning. We restricted our sample to Europe in order to make the task manageable and to increase the likelihood we could obtain unpublished details through our existing networks.

## Methods

Existing databases of birth cohorts (http://www.chicosproject.eu, http://www.enrieco.dk, http://www.birthcohorts.net) and Google searches were used to search for studies which met the following inclusion criteria (adopting those used in existing databases):

1) Study population based in Europe.

2) Cohort members were recruited ≤12 months of age including preconception.

3) Had a planned follow up period until cohort members reached at least adolescence.

4) The cohort collected more than biological samples, using for example questionnaires, interviews, educational assessments.

5) Included ≥200 mother-child pairs.

While the very smallest cohort were excluded from this group, 200 mother-child pairs is considered a small sample size for cohorts [27] and some smaller cohorts were included where they formed part of multi-cohort studies. Although our primary interest was in recent cohorts (i.e. 1990s onwards), no cut off date was considered appropriate a priori so no studies were excluded on this basis.

Three methods were used to collect data about the engagement practices of these cohorts: 1) information held on study websites and on the ENRIECO and birthcohorts.net websites; 2) further details included in published study documentation; and 3) direct contact with all cohorts. An excel spreadsheet was created to record information on the following topics:

• Location, topic and size of cohort.

• Details of Principle Investigator and home organisation of the cohort.

• Enrolment details (dates of enrolment, period of enrolment, age of child at recruitment, enrolment criteria, status of study).

• Data collection details (period of follow up, frequency of follow up, current age of cohort members, response rates at most recent follow up).

• Type of data collected, and from whom.

• Website (Existence and information provided).

• Communication methods with cohort members (e.g. newsletters, birthday cards, information about consent processes).

• Events held for cohort members.

• Consultation with cohort members.

• Views about the barriers to consultation/co-production.

All cohorts were approached at least twice (between October 2011 and March 2012) and asked to provide further information by email or to take part in a telephone interview. Interviews were audio recorded (but not transcribed), and information provided was entered directly into the Excel spreadsheet by the interviewer (DA).

Data were collected regarding response rates and attrition for each cohort (with the intention to compare rates between cohorts) but are not reported in detail here. Study attrition was seldom available, since many cohorts were ongoing and therefore participants could re-enter the study in the future, and reporting of response rates differed between studies [23]. We recorded whole sample response rates to the most recent wave of data collection where this was available, but this information was often missing. The different cohort contexts (size, current age, and geographic spread) together with missing data meant cross cohorts comparisons of retention and attrition was not possible or appropriate.

## Results

A total of eighty-four individual cohorts were identified. This represents 70 studies, since some cohorts were linked as a single study (for example the INMA cohorts consists of 7 cohorts from different regions of Spain [28]). We did not find any cohorts which we excluded on the basis of size alone. Sixty-one cohorts had project websites that could be accessed and which we used to obtain additional information about study conduct. Representatives of 32 cohorts (38% of those contacted) responded to emailed requests for additional information, 15 provided additional information via email and we interviewed representatives of 17 cohorts over the phone.

The recent growth in interest and investment in this study design is apparent in the numbers of studies identified. Ten cohorts were established prior to 1990, 23 in the period 1990-1999, 46 between 2000 and 2010 and 4 new cohorts since 2011. Most (n=73, 87%) of the studies collect data related to physical health only, but 11 also include measures of mental health, development, and social context more broadly. For most of these studies, response rates present a considerable problem, particularly over the longer term. Among the 23 cohorts that recruited during the period 1990-1999, response rates more than 10 years after enrolment varied between 13% and 84% for individual data rounds. Among ongoing studies, members could re-enter the study even after missing several data sweeps so it is difficult to say how many have to been lost to follow up.

Cohorts undertake a range of different activities to stay in touch with their cohort members, and to retain interest in the study. Table 1 summarises known engagement activities across the whole group of studies. Data is divided between those cohorts which provided further information, and those for which we only report what is reported in website and publications. Fifty-eight cohorts maintained a website (52 websites). However, these were often largely written in technical language and thus appear to be designed for the research community rather than the general public. For example, 32 websites provide links to publications, while 25 provide public-facing summaries of the study or findings. Seventeen project websites dedicate part of their website to some communication with participants, often space for parents to complete questionnaires online, information about how to stay in touch with the cohort, and descriptions of the studies in plain language. Three studies housed in the same research centre provide *separate* websites designed for cohort members and their families. These websites are informative, interactive, and provide information for cohort members and families, news, events and a wealth of other information. Five studies have web pages specifically for young cohort members themselves and one study provides a dedicated page for fathers. Six cohorts are known to use Facebook to stay in touch with their cohort members.

**Table 1:** Known engagement activities in birth cohorts

**Engagement activities**	**All cohorts**	**Non-responders (n=52)**	**Responders (n=32)**
Website	58	32	26
Newsletter, Bulletins & Magazines	20	3	17
Social Media	6	2	4
Games and competitions for children	3	1	2
Birthday cards	3	2	1
Health information (including individual health results)	4	2	2
Online questionnaires	4	3	1
Regular events	4	1	3
One-off events	1	-	1
Planning for greater engagement	3	3	-
Consultation with young people	5	-	5
Planning for greater consultation with young people	2	1	1
Consultation with parents	3	1	2
Space for feedback online or on questionnaires	8	1	7

Cohorts also actively stay in touch with their members; sending out newsletters with summaries of findings. We know of 20 studies which send information newsletters, or information about upcoming events or data collection waves, 3 which send cards (usually for birthdays), and 4 which give participants health advice. Five cohorts also hold events for families in their cohorts (such as parties) on an annual or occasional basis.

Webpages, newsletters and events are all useful engagement tools, but they are opportunities for study teams to speak to cohort members, not opportunities to listen to cohort members views and experiences. We therefore considered whether and how they consult with cohort members'. Since a minority of cohorts provided further information we are careful here to report known practice, acknowledging that study groups that didn’t respond to requests for information may undertake activities that they do not report publicly. The characteristics and activities of cohorts who responded to our request for further information are provided in Table 2. Study details have deliberately been generalised in order to protect anonymity for groups who provided information not in the public domain. Of these, 19 undertake no consultation with their study members although 2 said they would like to do so in the future and two had piloted questionnaires giving some opportunities for participant experience to be taken into consideration. Seven provide opportunities for participants to comment on the study; 6 use an open text box at the end of their questionnaires and one an online comment facility. Five use a range of approaches to consulting with research participants; 4 have consulted in an informal way with study parents (online forums, occasional or one-off meetings to comment on planned data collection). Just 2 of those responding to our queries have so far undertaken consultation with young people themselves. Two studies currently use young persons’ advisory groups, one comprising young people from within the cohort and the other young people of the same age who are not cohort members. These groups meet regularly and have provided important feedback on the conduct of the study including designing appropriate materials for obtaining informed assent, phrasing of some questions, and helped consider ways to improve retention of young people. We know that three cohorts who didn’t provide additional information to us also undertake consultation activities (one includes non-study parents in their advisory group, all three use parent forums or meeting) and 2 cohorts have stated plans to create opportunities to consult with study members in the future. Thus, we have been able to find eight cohorts that we know are consulting in some way with study members.

**Table 2:** Characteristics and consultation strategies among cohorts providing additional information

**Region**	**Focus**	**Date of first enrolment**	**Size of cohort (rounded to nearest 500)**	**Website**	**Other strategies to engage participants**	**Consultation with cohort members/parents**
Northern Europe	Narrow	Pre 1990	1,000	Yes	Photo exhibition	-
Northern Europe	Broad	Pre 1990	12,000	Yes	Package of individual health outcomes	-
Northern Europe	Narrow	Pre 1990	500	No	-	-
Northern Europe	Narrow	Pre 1990	9,500	Yes	Package of individual health outcomes	-
Northern Europe	Narrow	Pre 1990	1,000	Yes	Personal letter	Space is provided on questionnaires for feedback
					Engaging the Media	
					Dissemination through meetings	
Northern Europe	Narrow	1990-1999	1,200	No	Newsletters	-
Northern Europe	Narrow	1990-1999	<500	Yes	Personal letter	Space is provided on questionnaires for feedback
					Engaging the Media	
					Dissemination through meetings	
Northern Europe	Narrow	1990-1999	500	Yes	Personal letter	Space is provided on questionnaires for feedback
					Engaging the Media	
					Dissemination through meetings	
Northern Europe	Narrow	1990-1999	1,000	Yes		-
Northern Europe	Narrow	1990-1999	10,500	Yes & Facebook	Newsletters	One-off informal consult
Northern Europe	Broad	1990-1999	14,000	Yes including areas for participants & Facebook	Newsletters	Advisory group of young people
					Events	
						Online forum for parents
Northern Europe	Narrow	1990-1999	1,000	Yes	-	-
Northern Europe	Narrow	1990-1999	97,000	Yes including pages for children	Internet Questionnaires	Provide questions/space for feedback
Northern Europe	Broad	1990-1999	3,000	Yes	News articles & media	-
Northern Europe	Narrow	1990-1999	8,500	Yes & Facebook		One-off informal consultation used
Northern Europe	Narrow	1990-1999	<500	Under construction	Share media clippings at clinic visits	Open ended questions on survey for feedback purposes
Northern Europe	Narrow	2000-2009	<500	Yes	Personal letter	Space is provided on questionnaires for feedback
					Engaging the Media	
					Dissemination through meetings	
Northern Europe	Narrow	2000-2009	500	Yes	Personal letter, media,	Space is provided on questionnaires for feedback
					Dissemination through meetings	
Northern Europe	Broad	2000-2009	19,000	Yes		Not so far, but in planning
Northern Europe	Broad	2000-2009	1,000	Yes	Newsletters	Informal parent group
Northern Europe	Narrow	2000-2009	500	No	-	-
Southern Europe	Narrow	2000-2009	500	No	Newsletters	-
					Parties	
					Calendar	
					Birthday cards	
Northern Europe	Broad	2000-2009	2,000	Yes	Newsletters	-
Southern Europe	Broad	2000-2009	7,500	Yes & Facebook	Newsletters, Email, online questionnaires	
Northern Europe	Broad	2000-2009	8,000	Yes	Yearly event	Questionnaires piloted, but this is not considered consultation.
Northern Europe	Broad	2000-2009	1,500	Yes	Information event planned	-
Northern Europe	Broad	2000-2009	8,500	Yes	Newsletters	Advisory Group for Young People
Northern Europe	Broad	2000-2009	11,000	Yes	Newsletters	Advisory Group for Young People
Eastern Europe	Narrow	2000-2009	500	No	-	-
Northern Europe	Narrow	2000-2009	3,000	Yes including pages for children	Newsletters and media, online questionnaires used	-
Northern Europe	Broad	2010+	6,000	Yes	Yearly event	Questionnaires piloted, but this is not considered consultation.
Northern Europe	Broad	2010+	20,000	Yes including pages for children	-	-

We collected data on the location, focus, size, and start date of the cohorts (shown in Table 2). Newer cohorts were more likely to have websites, but since most cohorts maintain websites differences by decade are small. Only 1 of the smallest cohorts (≤500 members) maintained a website, but aside from this size and efforts to engage or consult with cohort members did not seem to be related. The age of cohorts did not seem to be related to how likely they were to engage with participants, with both some of the oldest and some of the youngest cohorts providing opportunities for feedback to the study teams.

Finally we asked study groups who agreed to be interviewed what made it difficult, or had discouraged them from greater use of consultation with their young participants. There were 5 barriers identified: concerns about impact on quality of research, ethical constraints, resource limitations, lack of importance, and difficulties experienced to date. Some study groups were concerned that further contact with participants would compromise research quality because of increased respondent burden and a risk of increased attrition or because of Hawthorne effects whereby the responses of their study group would be influenced by greater contact with the study team. In addition a number of ethical considerations were raised: some studies were bound by the original consents obtained which would not allow additional contacts with young participants; there was also a concern that any activity that might bring together study members would breach anonymity; and finally there were felt to be ethical constraints in working with the children independent of their parents. Resource limitations were a significant barrier; several study groups felt that they didn’t have sufficient resources for this work particularly where cohort members did not live near each other and where children of different ages were represented in a cohort. Related to lack of resource, was a belief that this kind of work might be interesting, but it either was not important or was not something they had considered, so it was not among their priorities. Where rates of retention were good, researchers felt there was no need for further engagement and thus the costs of doing so were not justified. Finally, some researchers felt that participatory work was difficult to get right, and past experience made them pessimistic that they would be able to achieve this.

## Discussion

This study shows that most European birth cohort studies undertake some activities to promote wider engagement in their ongoing work, but this is most often restricted to project websites. Very few actively and regularly consult with their members. A few others have facilities for participant feedback in response to data collection. This suggests that participant consultation is low among the priorities for this group as a whole, albeit that some are making concerted attempts to do so.

### Strengths and limitations

This study is the first to our knowledge that attempts to describe practice across a wide range of studies with similar methods on how they attempt to engage, inform and consult with their research participants. However, it has two significant limitations. Firstly, this study is restricted to practice in European cohorts, and so the description here excludes good practice elsewhere in the world. Secondly, we should be cautious to generalise from findings reported here, both because of the focus on European studies and because of the small number of studies for whom we have full information. Studies unknown to us, or who didn’t reply to our requests for information may have been undertaking participatory work which we don’t know about. However, we believe it is reasonable to assume that working in this way would increase the likelihood of responding to our requests for information, so it is likely we have described common practice among these studies. Within this small sample, the studies that undertook some form of consultation did not appear to differ from those that did not on their breadth of focus, time of inception, current age of participants, or size of cohort although larger cohorts more often had a formal approach to consultation.

### Comparisons to other studies

We know of no other study which has attempted to audit and describe practice in engaging young participants across a number of studies sharing a study design. One study surveyed methods for identifying, measuring and reducing attrition in 38 longitudinal studies with older adults (aged over 55 years) [29]. A range of activities similar to the ones identified here are reported (20 studies responded): information giving, newsletters, meetings, telephone calls, giving feedback about the study and summarising personal results, study websites, and incentives (fridge magnets, pens, money and calendars). Like us, they also found that variations in methods for reporting non-participation meant that cross study comparison was not possible, and that no data were available which suggested which strategies had been more successful [29].

A recent systematic review of strategies to increase retention in cohort studies shows that provision of financial incentives is the only strategy which has been shown to increase retention [23]. These authors note that other strategies are common, including reminders, allowing alternative modes of data collection, and a combination of several approaches but that the effects of these are seldom systematically recorded. They further comment on the possible impact of an expectation of being involved in a study for a long period of time [23]. In this context motivation to continue to take part might be influenced by participants’ feeling that the study is interesting, important, and pertinent to them. Documentation of methods to consult and engage with study members and any changes in response rates could usefully be recorded and reported in future.

Differing practice in gaining child assent or consent in six birth cohort studies has recently been described [11]. Several researchers have considered the views of child participants or their parents involved in longitudinal health studies about informed consent to participate. In 2 studies of the views of parents whose children were enrolled in a birth cohort study, Swartling and colleagues found that while many parents wanted their children to be informed they were somewhat cautious about allowing their children authority to make decisions and some did not feel children should provide consent [30,31]. Another study showed that when asked questions that were contextualized and concrete, child participants in a UK birth cohort study could understand and comment on ethical issues regarding consent for data storage and use [32,33]. Similarly, qualitative research with child participants in a Danish cohort was used to suggest ways in which tests and procedures could be made more pleasant and interesting for children [34].

Finally, we located 5 studies of the experiences of adult participants in longitudinal studies [35-38]. These studies suggest there is an appetite for more involvement in research decisions among some. In interviews with adult (aged 50 years) participants from the National Child Development Study (NCDS), a birth cohort, around a third wanted more feedback from the study about the findings, presented in a way that was useful to them. These studies identify a number of common levers and barriers to study retention. Reasons for staying in studies included the ‘prestige’ of being part of an important study [29], a commitment to the greater good by contributing to research [36], and a sense of belonging or loyalty to the study [35].

In contrast, questions and questioning that they viewed as intrusive [36], where the structure and purpose of survey items were unclear to respondents [37], disillusionment with past participation, or unpleasant past experiences with survey completion and a lack of commitment to the survey [38] caused study drop out. These findings confirm our belief that greater involvement in research planning is likely to increase study participation. Participants commented that the decision about what to observe in research is down to whether “*the powers that be”* look kindly upon this activity (P149, p. 13 [36]). They also comment on how poorly worded questions affected both data quality and created a barrier to participation:

*In the last one there was a question about how often do you see your children, …I had to put like I only saw him three times a year or something, which it was wrong, because I see him for long times when I see him….he’s at university …. but the form wouldn’t allow that. And I feel quite upset about it really though* P351] [36].

### Implications for cohort research groups

This study suggests that while most birth cohorts put in place methods to keep participants informed about the research they are involved in through study websites and newsletters, very few are creating opportunities to hear from their participants. Where cohort members are still very young consultation with their parents could be undertaken, but seldom is. Methods currently used range from simply providing the opportunity to enter text responses after completing a round of data collection to informal and formal mechanisms for consulting with participants. The success of these methods in either increasing participant engagement and retention, data quality, or in influencing research conduct have not been assessed to our knowledge. While calls for greater engagement in the research process are often value based and therefore do not rely on demonstrating efficacy, data on the relative success of different methods would be valuable and research documenting impact would be important.

In the absence of evidence to support selection of particular methods, it may be useful for those cohorts with younger members or those who undertake less participation to learn from those cohorts which undertake more engagement in planning for the future. This would allow, for example, anticipating and preparing for the transition from parent consent for infants, to assent and consent from children themselves.

The barriers to increased participation raised by the cohort researchers in this study are not unique to this study design. Barriers to involving children and young people in research are well outlined (and countered) by Kellett in her account of the work of the Children’s Research Centre. She reports concerns about children’s’ skills and competency, time and resource constraints, and worries that their participation will be meaningful [9]. Involving young people can require skills that are more commonly associated with a participation officer than a researcher. As well as practical constraints, the gap between the knowledge systems of research and participation are known to create barriers to meaningful collaboration [8,39,40]. However, these barriers have been addressed in non-cohort studies [7,9,20,21,41] and examples of innovative practice showcase the output of young people working as researchers [42].

Through EUCCONET [43], European researchers are beginning to share practice on participant retention in birth cohorts, but not yet on participant consultation. Practical guidance exists for increasing involvement [18,19] and lists of resources are provided by UNICEF [44] and INVOLVE (a national advisory group that supports greater public involvement in UK health research) [45]. Changes need to be meaningful, but do not need to be large; current efforts to retain study members using Facebook [46-48] and family events could be used to create opportunities for contributions from cohort members without challenging some of the concerns of researchers regarding ethics and Hawthorne effects. Development of methods in this field is timely and would facilitate cooperation with existing and new major projects.

## Conclusions

This study identified varied practice in consultation with parent and child cohort members; most send information to participants and reward participation, a few have feedback mechanisms for cohort members, very rarely there are opportunities for members to contribute to research planning through consultation. Barriers to increased consultation were identified as fears for research quality, ethical concerns, lack of resources, poor past experiences, and a belief this wasn’t necessary. These barriers have been overcome in some cohorts and in other fields, and these provide examples of how more consultation could be undertaken within the scope of funding and resources. The large increase in cohorts initiated in the last 10 years means that there are a large number of children who are soon to be old enough for consultation to take place.

### Consent

Email requests for information informed researchers that in replying with information they provided consent for us to use this information anonymously. Verbal informed consent was obtained from the researchers interviewed over the phone for this project.

## Competing interests

The authors declare that they have no competing interests.

## Authors’ contributions

PJL conceived of, and obtained funding for the study. All authors designed the study. DA collected and analysed the data overseen by PJL, and PJL wrote the first draft of this manuscript. All authors read, reviewed and approved all versions of the manuscript.

## Pre-publication history

The pre-publication history for this paper can be accessed here:

http://www.biomedcentral.com/1471-2288/13/56/prepub
